# Azacitidine and lenalidomide combination: a novel relapse prophylaxis regimen after allogeneic hematopoietic stem-cell transplantation in patients with acute myeloid leukemia

**DOI:** 10.3389/fimmu.2023.1182251

**Published:** 2023-06-22

**Authors:** Yimei Feng, Ting Chen, Yun Zhang, Han Yao, Ping Wang, Lu Wang, Kaniel Cassady, Zhongmin Zou, Yuqing Liu, Lu Zhao, Lei Gao, Xi Zhang, Peiyan Kong

**Affiliations:** ^1^ Medical Center of Hematology, The Xinqiao Hospital of Army Medical University, Chongqing, China; ^2^ Regeneron Pharmaceuticals, Tarrytown, NY, United States; ^3^ Department of Chemical Defense, School of Military Preventive Medicine, Army Medical University, Chongqing, China

**Keywords:** allo-SCT, azacitidine, lenalidomide, maintenance therapy, AML

## Abstract

**Introduction:**

While allogeneic hematopoietic stem cell transplantation (allo-HSCT) can be a curative regimen for acute myeloid leukemia (AML), relapse of AML remains a serious risk post-transplantation. Once relapsed, salvage options are limited and management of AML is difficult. Here we designed a prospective study to examine the efficacy and tolerability of maintenance therapy with azacytidine (AZA) plus low-dose lenalidomide (LEN) to prevent relapse after allo-HSCT for AML patients (ChiCTR2200061803).

**Methods:**

AML patients post-allo-HSCT were treated with AZA (75 mg/m^2^ for 7 days), followed by LEN (5 mg/m^2^, day 10-28), and a 4-week resting interval, which was defined as one treatment cycle. A total of 8 cycles was recommended.

**Results:**

37 patients were enrolled, 25 patients received at least 5 cycles, and 16 patients finished all 8 cycles. With a median follow-up time of 608 (43-1440) days, the estimated 1-year disease free survival (DFS) was 82%, cumulative incidence of relapse (CIR) was 18%, and overall survival (OS) was 100%. Three patients (8%) had grade 1-2 neutropenia without fever; one patient developed grade 3-4 thrombocytopenia and minor subdural hematoma; 4/37 patients (11%) developed chronic GVHD with a score of 1-2, without requiring systemic treatment; No patient developed acute GVHD. After AZA/LEN prophylaxis, increasing numbers of CD56^+^NK and CD8^+^ T, and decreasing of CD19^+^ B cells were observed.

**Discussion:**

Azacitidine combined with low-dose lenalidomide was observed to be an effective relapse prophylaxis option after allo-HSCT in AML patients, and can be administered safely without significantly increasing the risk of GVHD, infection and other AEs.

**Clinical Trial Registration:**

www.chictr.org, identifier ChiCTR2200061803.

## Introduction

Allogeneic hematopoietic stem cell transplantation (allo-HSCT) plays an important role in the treatment of intermediate and high-risk acute myeloid leukemia (AML). However, 30-80% of patients are destined to relapse following allo-HSCT (1). Relapse is now the major cause of treatment failure in AML patients after HSCT. Following relapse, either a second transplantation or donor lymphocyte infusion (DLI) can be used to treat relapsed disease, but the efficacy is unsatisfactory at present (2, 3). Early intervention during AML relapse may improve patient outcomes (4), while the holy grail post-allo-HSCT remains the prevention of relapse. With the advent of new drugs, there is an increased focus on the optimization of prophylaxis regimens after transplantation to improve the long-term survival of AML patients.

Recently, two drugs azacitidine (AZA, hypomethylating agent/HMA) and lenalidomide (LEN, immunomodulator/IMiD), have each been shown to possess significant anti-leukemic activity against AML (5, 6). In relapsed patients post-allo-HSCT, 15-20% of patients achieved a complete remission (CR) when receiving AZA treatment, but median time to CR was 108 days (7), highlighting a potential opportunity to optimize AZA therapy post-allo-HSCT. Craddock et al (8). conducted a dose-finding study of LEN administered in combination with AZA in relapsed AML patients after transplantation. The maximum tolerated dose of LEN was 25 mg. In the entire patient set, the median OS of the responders was better than 10 months’ median OS observed in non-responders significantly (9). However, because LEN can activate NK cells and T cells, it has been reported that the use of LEN may cause severe graft-versus-host disease (GVHD) in patients after allo-SCT (10, 11). On the other hand, AZA can accelerate reconstitution of T regulatory cells after transplantation, induce immune tolerance, and reduce the risk of GVHD (12, 13). Therefore, we hypothesized that combination of AZA and low-dose LEN could simultaneously provide anti-leukemic activity after transplantation without increasing, overall, the risk of severe GVHD. Here we describe a prospective study to examine the efficacy and tolerability of maintenance therapy with AZA plus low-dose LEN to prevent relapse after allo-HSCT for AML patients.

## Patients and methods

### Prophylaxic plan

**Figure 1:**
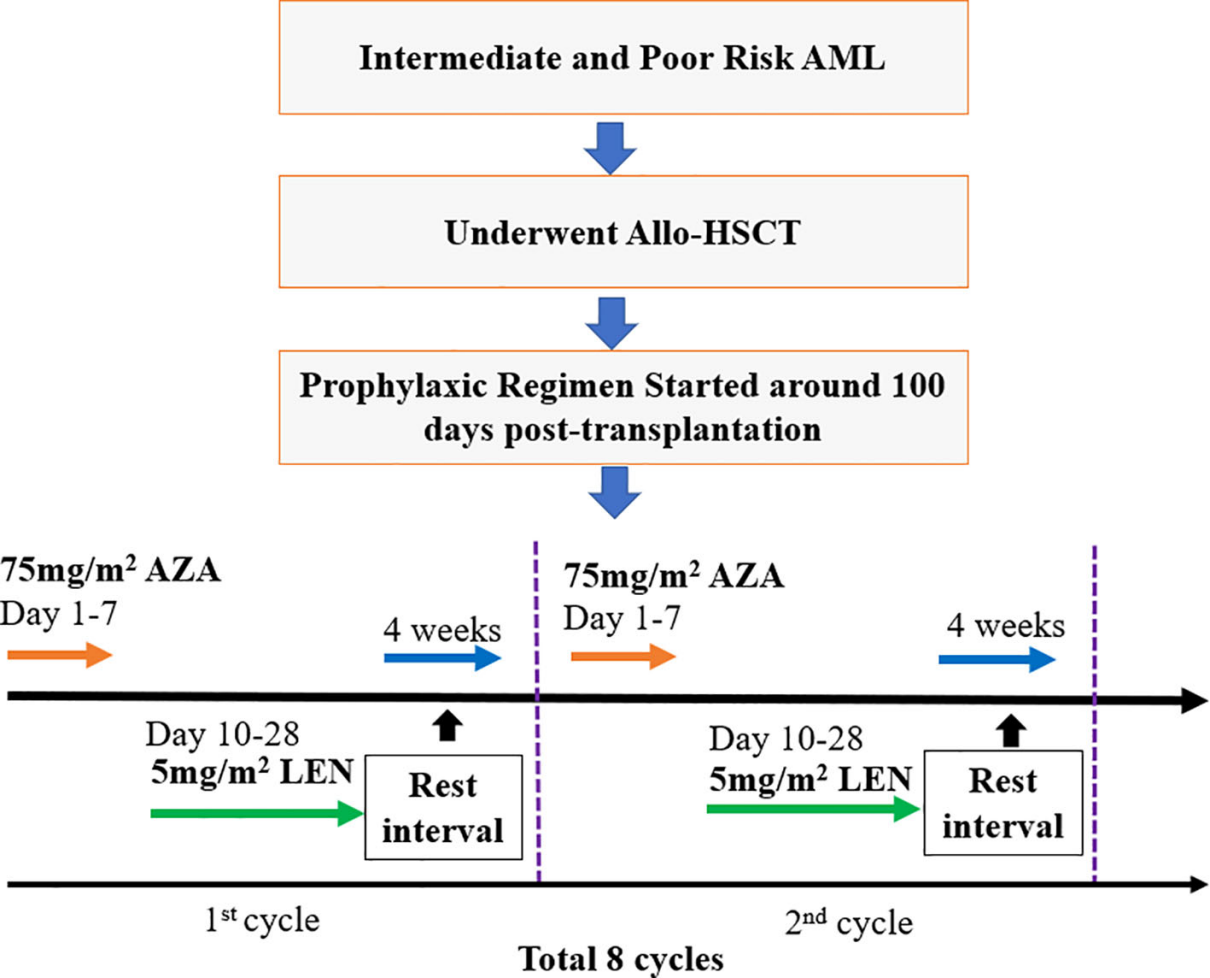
Patient enrollment flow chart and prophylaxis regimen.

This clinical trial (ChiCTR2200061803 at the Chinese Clinical Trial Registry, www.chictr.org) was initiated by our medical center (Xinqiao Hospital) as a prospective, open-label, single-arm trial design of LEN in combination with AZA in AML patients who received allo-HSCT. The enrolled patients received AZA treatment (75 mg/m^2^ for 7 days) on day 1, approximately 100 days post-HSCT, followed by oral administration of lenalidomide from the day 10 (5 mg/m^2^/day) to day 28, followed by a one-month rest interval, which was defined as one cycle. In total, 8 cycles were recommended as a complete treatment course. Patient enrollment flow chart and a diagram of the prophylaxis regimen is summarized in **Figure 1**.

Patient inclusion criteria: (1) Relapsed and refractory (R/R) AML patients who received allo-HSCT (for criteria of R/R AML refer to Chinese guidelines for the diagnosis and treatment of relapsed/refractory acute myelogenous leukemia (14)); (2) Successful hematopoietic reconstitution after transplantation and without GVHD (3). The acute GVHD (aGVHD) below grade 2, and/or chronic GVHD (cGVHD) below score of 2, who don’t need systemic treatment for GVHD; (3) Patients without severe infection or organ failure after transplantation.

Patient exclusion criteria: (1) Patients who have already relapsed (including molecular and cytological relapses) at the beginning of this protocol after transplantation; (2) Patients with grade 2 or above acute GVHD or chronic GVHD with a score of more than 2; (3) Those who were allergic to the study drugs; (4) Those who researchers assessed as unfit.

### Clinical outcome assessment

Leukemia relapse monitoring was performed by assessing bone marrow once a month in the first 6 months post-HSCT and thereafter every 2^+^ months, depending on the patient’s condition. The frequency of BM examination could be increased if necessary. Flow cytometry (FCM) and real-time quantitative polymerase chain reaction (qRT-PCR) were used to monitor minimal residual disease (MRD). The MRD monitoring interval was the same as that for the bone marrow biopsy. MRD positivity was defined as >0.01% of cells with leukemia-associated aberrant immune phenotypes in the bone marrow (15), or transcript level ≥0.001% for leukemia-related genes, including AML1/ETO, FLT3-ITD, DNMT3A, MLLAF9, MLL/AF4, etc. Patients were scored as MRD positive if they had 2 consecutive positive results using FCM or PCR or both FCM and PCR were positive in a single sample. Regimen-related hematological toxicity was monitored once a week in the outpatient clinic, including routine blood, liver, and kidney function. Analysis of lymphocyte subsets was also performed for the enrolled patients, including T, B, and NK cell analysis. The Adverse Events assessment is according to the National Cancer Institute Common Terminology Criteria 4.03.

### Study outcomes and statistical analysis

The follow-up time point of the article ended in May 15, 2023. The primary endpoint was incidence of relapse; secondary endpoints included overall survival (OS), disease-free survival (DFS), and safety of the medication regimen. OS was measured from the time of prophylaxis intervention to death from any cause. DFS was defined as the time from prophylaxis intervention to relapse (including molecular and cytological relapse), progression, or death. Cumulative incidence of relapse (CIR) was defined as the time from prophylaxis to disease relapse or progression. Non-relapse mortality (NRM) was measured from the time of transplantation to death from any cause other than disease relapse or disease progression. The one-way ANOVA test was used to analyze the proportional difference of lymphocyte subsets among different timepoints. The Kaplan–Meier estimator was used to estimate the survival curves. *P* < 0.05 was assigned as statistical significance. Graphpad prism (8.0) was used to carry out all the above statistical analyses and the drawing of survival curve.

## Results

### Patient characteristics

A total of 37 AML patients were enrolled in the study, including 21 males (57%) and 16 females (43%), with a median age of 31 years (4-58years). Disease status before transplantation for all patients was either CR (complete remission) or PR (partial remission), CR with MRD negative accounted for 81% (30/37), CR morphologically but MRD positive accounted for 14% (5/37). Two patients (5%) achieved PR before transplantation. The MRD of all patients was evaluated by flow cytometry, and 8 patients were fusion gene positive at first diagnosis as detected by PCR. The risk stratification of the disease was as 9 intermediate risk patients (24%) and 28 high risk patients (76%). Donors were classified as 7 HLA-identical sibling donors (19%), 13 Haplo-identical donors (35%), and 17 unrelated donors (46%). The median chemotherapy cycle before transplantation was 4 cycles (3-8 cycles). The median CD34^+^ cells numbers for transplantation was 7×10^6^/kg (2.86-11.57). The median time to neutrophil engraftment (≥0.5×10^9^/L) after transplantation was 15 (10-23) days, while that of platelet engraftment (≥20×10^9^/L) was 16 (11-27) days (**Table 1**).

**Table 1:** Patient Characteristics.

Patient Characteristics	No. (%) or (range)	
**Total patients**	37	
**Median age at transplantation, y (range)**	31	(4-58)
**Gender** **Male** **Female**	21 16	57% 43%
**Disease status at transplant** **CR** **MRD-** **MRD+** **PR**	35 30 5 2	95% 81% 14% 5%
**Number of complete remissions** **CR1** **CR2** **Others**	28 7 2	76% 19% 5%
**MRD detection method** **Flow cytometry** **PCR**	37 8	100% 22%
**High-risk factor** **WBC > 100 × 10^9^ at diagnosis** **Inferior cytogenetic aberrations** **Molecular characteristics with poor prognosis** **Combined with CNSL** **Extramedullary infiltration** **History of MDS /MPN** **Relapse after the first transplantation**	1 5 20 1 1 4 2	3% 14% 54% 3% 3% 11% 5%
**Cytogenetic risk group**		
**Favorable risk**	0	0
**Intermediate risk**	9	24%
**Poor risk**	28	76%
**Donor type**		
**Haplo-identical**	13	35%
**HLA-identical sibling**	7	19%
**Unrelated donor**	17	46%
**Median chemotherapy number before transplantation**	4	(3-8)
**Median CD34^+^ cells at transplant, 10^6^ (range)**	7	(2.86-11.57)
**Median days of neutrophils≥0.5×10^9^/L after transplantation**	15	(10-23)
**Median days of platelets≥20×10^9^/L after transplantation**	16	(11-27)
**Median follow-up time (range)**	608	(43-1440)

CR, complete remission; PR, partial remission; MRD, minimal residual disease; PCR, polymerase chain reaction; CNSL, central nervous system leukemia; MPN, Myeloproliferative Neoplasms.

### Clinical outcomes: Safety, relapse, OS, and DFS

Sequential AZA and LEN prophylaxis therapy was safe and well tolerated. The median number of prophylaxis cycles was 7 (range, 1-8 cycles), while 25 patients received at least 5 cycles, and 16 patients finished all 8 cycles. The adverse events included a total of 3/37 patients (8%) who displayed grade 1-2 neutropenia while none had agranulocytosis with fever; one patient with grade 3-4 thrombocytopenia developed minor subdural hematoma, and recovered safely after symptom-oriented treatment; 5/37 patients (14%) exhibited mild rash with pruritus; 4/37 patients (11%) developed cGVHD following treatment with AZA/LEN with a score of 1-2 not requiring systemic treatment cGVHD; No patient developed symptoms consistent with aGVHD. The NRM was zero.

**Figure 2:**
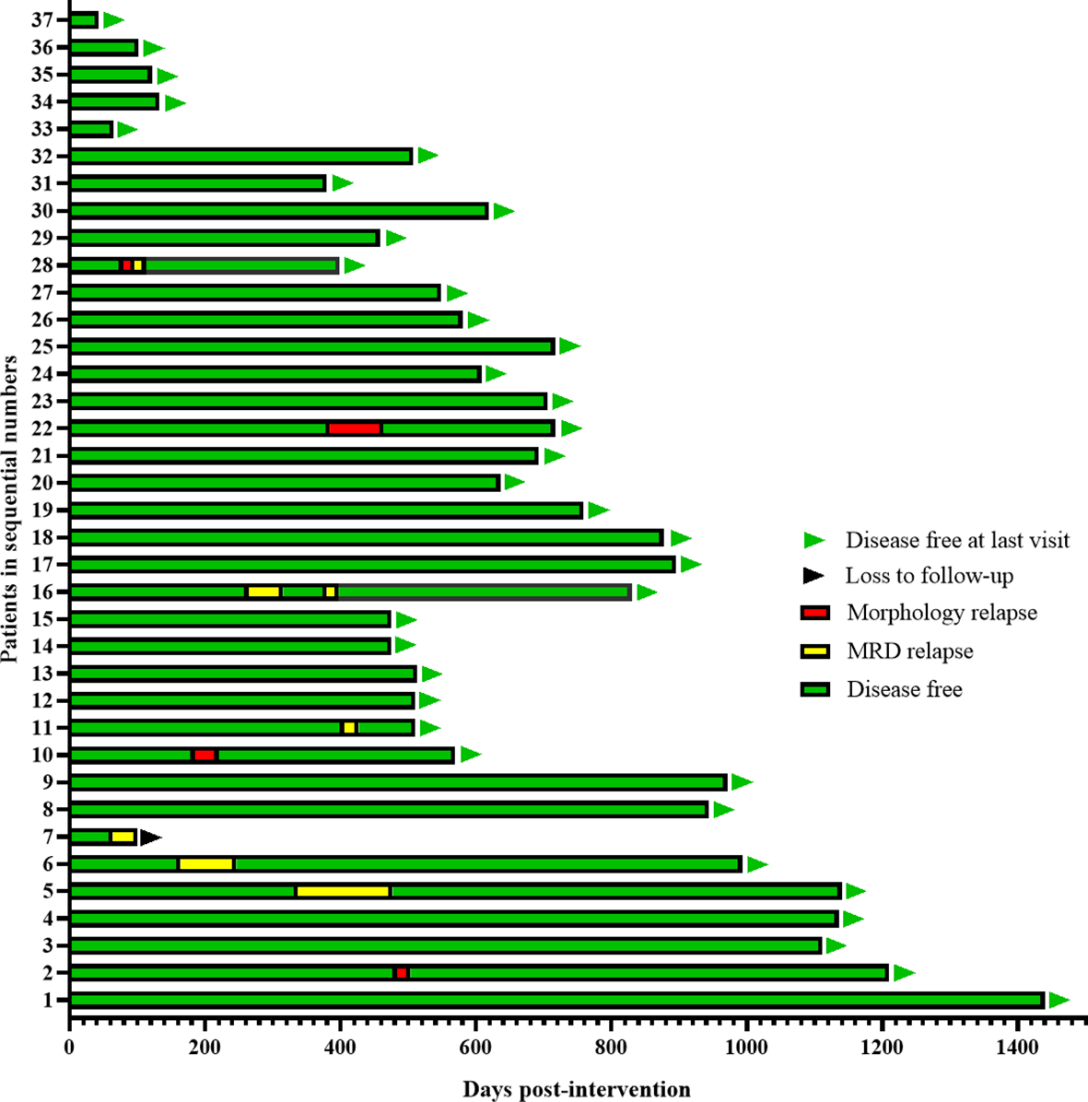
Clinical outcome of prophylaxis maintenance after transplantation.

**Figure 3:**
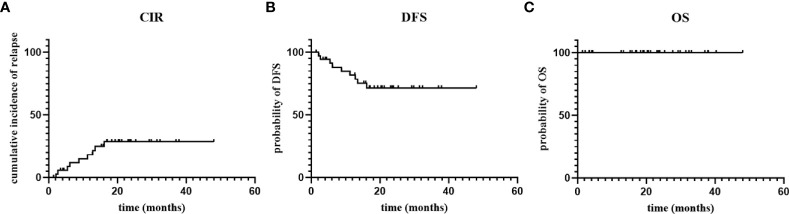
The cumulative incidence of relapse (CIR), disease-free survival (DFS) and overall survival (OS).

With a median follow-up time of 608 (43-1440) days, Five patients (14%) had molecular relapse but three patients became MRD negative again after continued treatment with the AZA/LEN regimen, one attaining MRD negativity after DLI and venetoclax treatment, and one patient was lost to follow-up. There were 4 patients who developed cytological relapse, received other subsequent treatment and were still alive with disease (**Figure 2**). Calculating results demonstrated that the estimated 1-year CIR was 18%,1-year DFS was 82%, and OS was 100% (**Figure 3**).

**Figure 4:**
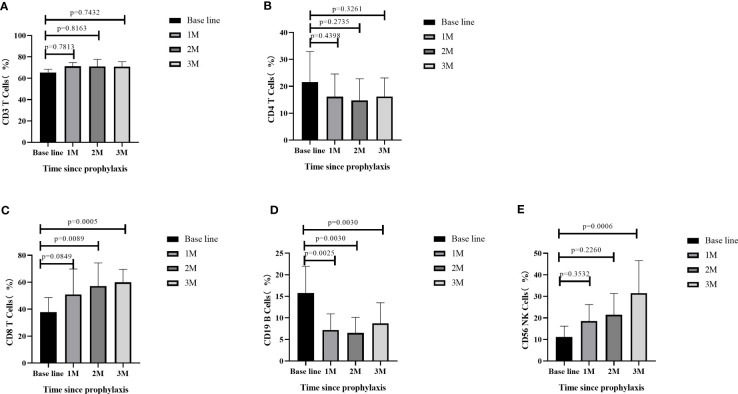
Dynamics of lymphocyte subsets after prophylaxis.

Flow cytometry analysis was performed to measure the lymphocyte subsets at four different timepoints: AML patients post-allo-HSCT prior to AZA/LEN prophylaxis (baseline), following the first month after receiving AZA/LEN prophylaxis (1M), the second month after receiving AZA/LEN prophylaxis (2M), and the third month after receiving AZA/LEN prophylaxis (3M). We measured immune cell subsets in some of the patients and observed a significant change in the proportions of CD4^+^ T, CD8^+^ T, CD19^+^ B and CD56^+^ NK cells subsets after prophylaxis. While the percentage of CD3+ T cells did not change following prophylaxis with AZA/LEN, both CD8^+^T and CD56^+^NK cell percentages in these patients were increased in the periphery after AZA and LEN intervention. In contrast, CD19^+^ B cells decreased dramatically following AZA/LEN administration (**Figure 4**).

## Discussions

Leukemia relapse after allo-HSCT is the major cause of treatment failure for AML patients. Once relapsed, salvage treatments are limited. Therefore, a prophylaxis regimen that can prevent AML relapse post-HSCT is needed. Currently, the most common prophylaxis regimens for preventing relapse after allo-HSCT include tyrosine kinase inhibitors (TKI), HMAs, immune checkpoint inhibitors (ICI), donor lymphocyte infusions (DLI), and immunotherapy. In this study, azacitidine combined with lenalidomide (AZA/LEN), as a novel relapse prophylaxis option, acquired satisfactory CIR, DFS and OS rate, and importantly evoked less adverse events. This regimen provided an effective and safe relapse-preventive therapy for AML patients after allo-HSCT.

The impact of AZA in the post transplantation setting is controversial (16). Several studies have previously reported AZA as an effective prophylactic treatment after allo-HSCT (17–19). In contrast, other studies failed to demonstrate a beneficial impact of post-transplantation prophylactic AZA on patient outcomes (20, 21). Moreover, AZA may lead to severe cytopenia, especially in early post-transplant period in frail patients (22). Lenalidomide, an immunomodulatory drug, is another candidate for potential maintenance therapy after allo-HSCT (23). It has broad effects on cytokines, immune cells, and angiogenesis, such as increasing the activity of NK cells and cytotoxic T cells and playing a specific role in the GVL (graft versus leukemia) effect. However, it may also enhance aGVHD even at modest doses of 5 to 10 mg^10^ (24). A striking observation in this study was the tolerability of LEN with acceptable rates of GVHD in patients after allo-HSCT. One possible explanation is the timing of administration, as we administered LEN 100 days post-allo-HSCT, outside the window of aGVHD. It could also be related to the combination with AZA, which may augment T-regulatory cell expansion and decrease the risk of GVHD (9, 25). Additionally, HMA application after transplantation can reactivate tumor suppressor genes and re-expression HLA-DR in tumor cells (26), and is associated with an increase in the proportion of WT1 positive cytotoxic T lymphocytes (WT1^+^CTL) (27). Of note, combined AZA/LEN therapy has been used as successful salvage therapy in patients who had relapsed post-transplantation. In one example, 7 of 15 (47%) patients who received at least three cycles of LEN/AZA salvage achieved a major clinical response as 3 CR, 3 CR with incomplete blood count recovery, and 1 PR patient) (8).

Our strategy in this paper was to move AZA/LEN administration earlier post-allo-HSCT to assess impact on preventing relapse after transplantation in AML patients, rather than as a salvage treatment after relapse has occurred. Our study enrolled 37 patients, with a median follow-up of 608 days. Under the intervention of AZA/LEN, only 9 cases relapsed, and the remaining 28 cases survived with disease-free in the observation period. The results showed 82% of 1-year DFS, 18% of CIR, 100% of OS, 0% aGVHD and 11% of cGVHD, which was superior to DLI based regimen with 1-year DFS 58-62.5%%, 74.5-78.8% of OS and aGVHD 7-8.7% (28–30). Notable, we defined the starting time of OS, DFS, and CIR from the implementation of prophylaxis, with the aim of better reflecting the clinical efficacy of this prophylactic measure, because there are too many interference factors such as chemotherapy and transplantation which cannot simply reflect the effectiveness of prophylaxis, if started from diagnosis or transplantation. Anyway, we re-analyzed the outcomes from time of transplantation, and found that the estimated 1-year CIR was 12%, and 1-year DFS was 88% (**Supplementary File**), that are superior than statistical data of which from the beginning of prophylaxis. It is speculated that benefited from the dual effects of transplantation conditioning regimen and prophylaxis regimen. Meanwhile, the state of MRD before transplantation is an important factor determining relapse after transplantation, and we found that for patients with MRD positive before transplantation, maintenance therapy earlier after transplantation can help better reduce relapse, so we are currently conducting a phase 2 study, using the AZA+LEN regimen in advance within 100 days, when hematopoietic reconstruction after transplantation. Although this paper is limited to a single arm and small size study, we demonstrated that AZA can be safely administered post-SCT in combination with LEN for prophylaxis, which seems to be associated with a higher anti-leukemic activity.

The detailed mechanism of anti-leukemic activity of AZA+LEN remains to be explored. We assessed lymphocyte subsets, and observed a significant increase in the proportions of CD8^+^ T, and CD56^+^ NK cells subsets after prophylaxis. It is speculated that cytotoxic CD8^+^ T cells and NK cells activation can augment GVL effect to restrain relapse. AZA has previously been shown to up-regulate tumor antigen expression on AML blasts and can also induce a CD8^+^ T-cell response post-allograft (19, 31), whereas post-allograft lenalidomide induces strong NK cell-mediated anti-tumor activity (23). Most reports mentioned that LEN may cause GVHD, but there were also opposite reports recognizing LEN as a useful prophylactic agent for aGVHD-induced mortality through the inhibition on lymphocyte migration to the gastrointestinal tract in mice model (32). In our study, the number of CD19^+^ B cells ratio decreased dramatically after prophylaxis. However, the impact of this reduction on relapse and GVHD pathogenesis, such as reduction in autoantibodies, must be further explored. Additionally, detection of other immune subsets (such as Tregs, Bregs, MDSC, etc.) and other biomarkers such as cytokines (chemokines, inflammatory factors, etc.) will be further explored in future studies.

In conclusion, the results of the current study suggest that maintenance treatment with AZA and low-dose LEN combination introduced around 100 days after allo-HSCT is surprisingly efficacious, with an acceptable toxicity profile and impressive long-term disease control. In addition, this treatment did not have much impact on cGVHD, and its impact on GVL requires further clarification with more indicators. However, this is only a small sample, single-arm, one center clinical study, and definitely is worthy of further investigation in a larger cohort with longer follow-up period.

## Data availability statement

The raw data supporting the conclusions of this article will be made available by the authors, without undue reservation.

## Ethics statement

The studies involving human participants were reviewed and approved by the ethics committees of Xinqiao Hospital. Written informed consent to participate in this study was provided by the participants’ legal guardian/next of kin. Written informed consent was obtained from the individual(s), and minor(s)’ legal guardian/next of kin, for the publication of any potentially identifiable images or data included in this article.

## Author contributions

YF and PK contributed to the design and conceptualization of the research, design of data analyses, interpretation of data, collection of data, and writing of the manuscript. TC, LW, HY, YL, LZ and LG managed patients and reviewed this paper. YZ and PW collected FCM data and draw the figures. KC, ZZ, and XZ edited this review. XZ and TC funded the work. All authors contributed to the article and approved the submitted version.
