# Supplementary material for: Azacitidine and lenalidomide combination: a novel relapse prophylaxis regimen after allogeneic hematopoietic stem-cell transplantation in patients with acute myeloid leukemia
Source: Front Immunol. 2023 Jun 22;14:1182251. doi: 10.3389/fimmu.2023.1182251 (PMC10332158; doi:10.3389/fimmu.2023.1182251)
Supplement: Supplementary file 1 [file DataSheet_1.docx]

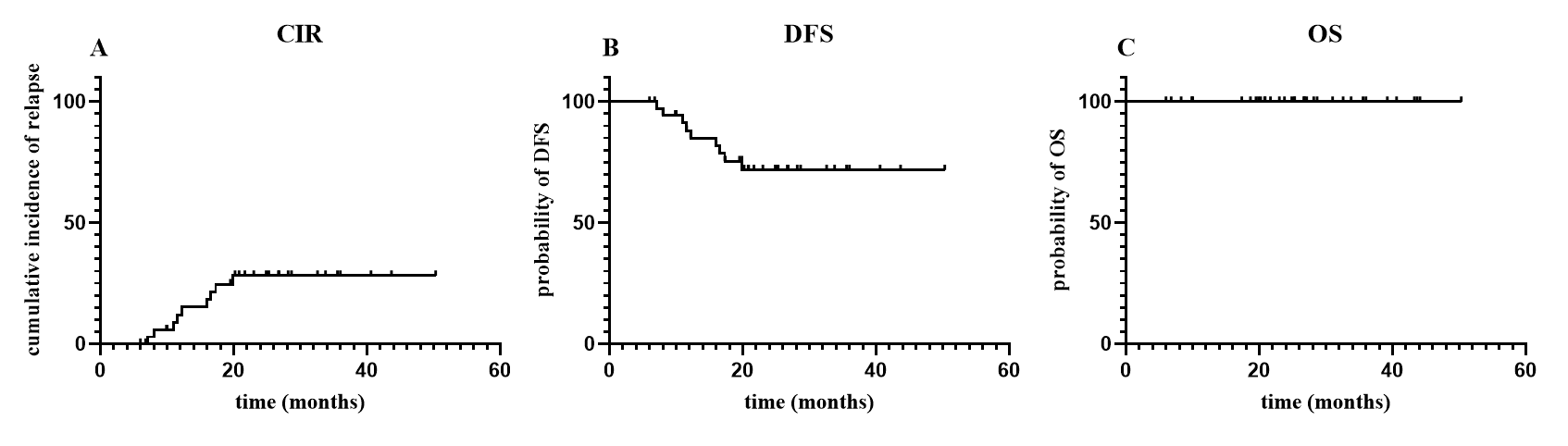


**The cumulative incidence of relapse (CIR), disease-free survival (DFS) and overall survival (OS) from the beginning of transplantation**
